# Voluntary Work for Military Hospitals

**Published:** 1916-01-29

**Authors:** 


### VOLUNTARY WORK FOR MILITARY HOSPITALS.

### A Bournemouth Workshop.

To say that Bournemouth possesses an organisation for making hospital appliances that fall within the scope of the wood-worker would be simply to make a statement which is equally true of other towns up and down the country. There are, however, one or two features in the Bournemouth scheme which are claimed to be original.

The organiser of the work is the Instructor of Carpentry and Joinery at the Municipal College.

Starting out with the idea that the whole of the scheme should be voluntary, he first obtained permission from the Education Committee to use one of the well-equipped wood-work rooms at a Manual Training Centre in a residential part of the town. The Committee gave the use of the room, the tools, and the light free of cost for one evening per week.

Invitations were then sent out through the secretary of the trade union to the carpenters and joiners in the locality to devote one evening per week to this useful work; old students of the technical classes, and others not connected with the trade, but who had some experience of wood-work, were also invited. On the whole, the response was satisfactory, and a band of upwards of a dozen meet each week for three hours' hard work, the organiser acting as "shop foreman," and giving to each worker the class of work for which he is best suited; no one desires to pick and choose, and all are willing to dispose of their labour in such a manner that the best results may be obtained.

### Where the Wood Comes From.

Practically all the material has been obtained on the "voluntary system"; two local firms who are working on Government contracts gave wood, some of which was in sizes too small to be of much value to them, but proved useful for this work; the glue, glass-paper, nails, etc., were also given by local tradesmen, so that the whole of the organisation was set up without raising any funds. The generosity of builders, however, is not unlimited, and other means of providing wood had to be considered. One of the enthusiastic workers suggested that grocers' boxes were generally made of wood. An appeal to the local tradesmen proved this to be correct, and some really serviceable material was found in that way. Even the drapers were tapped, and the boards which come out from the rolls of dress material were almost ready-made splints. The three-ply wood of which tea-chests are made is strong and light, and makes excellent bottoms for trays—a little scraping, plenty of sand-paper, and some stain will work wonders in the hands of a practical man; and, considering the temporary nature of the hospitals in which these articles will be used, this class of goods is very suitable.

When the work was started early in October local requirements were first considered. A Red Cross hospital was extending its premises, and accepted six shaped back-leg splints with movable foot-piece, six pairs shaped wrist splints with plain splints to match. The work being limited to one night per week, and the number of workers small at first, this occupied about three or four weeks, but as fresh workers offered their services the work extended. The St. John Ambulance Association have recently opened a hospital, and are being supplied with folding-screens and bed-tables, and there is talk of fittings for an operating theatre. The work has also spread to hospitals in France where English matrons are in charge. Letters are received in which they mention the articles that would be most useful to them, and it is satisfactory to note that splints do not seem to be in great demand, the chief cry being for bed-tables (that will fold for packing), trays for carrying round dressings, knife-boxes, etc. The workers are in this way endeavouring to supply a definite demand and are not making things with the uncertainty as to whether they will ever be used.

In conclusion, it should be mentioned that the boys from the elementary schools who attend the Wood-work Centre during the day are delighted when they are given the opportunity of making any of the simpler articles or even parts of articles that the men will finish in the evening.

As games are in great demand, the boys have been busy making sets of draughtsmen and boards to send as gifts to one of the hospitals "somewhere in France." These may be more crude than the shop-purchased game, but there is a fund of good-will behind them that will surely be appreciated. The boys, many of whom are Scouts, prove very useful in collecting material and delivering goods locally in their out-of-school hours. This work will continue as long as there is a demand, but no one will be sorry when the cause of the demand has been removed.

